# Cultivable Fungi from Amazon River Dolphins Engaged in Wildlife Ecotourism in the Anavilhanas National Park, Brazil

**DOI:** 10.1155/2024/1267770

**Published:** 2024-04-23

**Authors:** Marla Jalene Alves, Fernanda Rodrigues Fonseca, Layssa do Carmo Barroso, Érica Simplício de Souza, Marcelo Derzi Vidal, Ani Beatriz Jackisch-Matsuura, João Vicente Braga de Souza, Salvatore Siciliano

**Affiliations:** ^1^Laboratório de Diversidade Microbiana da Amazônia Com Importância Para a Saúde, Instituto Leônidas e Maria Deane/Fiocruz Amazônia, Manaus 69057-070, AM, Brazil; ^2^Laboratório de Modelagem em Estatística, Geoprocessamento e Epidemiologia, Instituto Leônidas e Maria Deane/Fiocruz Amazônia, Manaus 69057-070, AM, Brazil; ^3^Laboratório Central de Saúde Pública, Fundação de Vigilância em Saúde do Amazonas, Manaus 69093-018, AM, Brazil; ^4^Escola Superior de Tecnologia, Universidade do Estado do Amazonas, Manaus 69050-020, AM, Brazil; ^5^Centro Nacional de Pesquisa e Conservação da Sociobiodiversidade Associada a Povos e Comunidades Tradicionais, Instituto Chico Mendes de Conservação da Biodiversidade, São Luís 65020-270, MA, Brazil; ^6^Laboratório de Micologia, Instituto Nacional de Pesquisas da Amazônia, Manaus 69067-375, AM, Brazil; ^7^Departamento de Ciências Biológicas, Escola Nacional de Saúde Pública/Fiocruz, Rio de Janeiro 21041-210, RJ, Brazil

## Abstract

Amazon River dolphins are an important flagship species in the Anavilhanas National Park, Brazil, where they interact with visitors. This study aimed to quantify and identify fungi isolated from dolphin skin and oral samples and their surrounding environment in this unique ecosystem. Samples were collected from three dolphins and water samples from Flutuante dos Botos and the Novo Airão city harbor. Fungi were isolated using culture media and identified through micromorphology assays and ITS region sequencing. Oral swab samples resulted in culture of *Trichosporon montevideense* and *Exophiala dermatitidis*. Skin samples from one dolphin revealed *Toxicocladosporium irritans* and *Diaporthe lithocarpus*. Water samples exhibited higher fungal counts and diversity, with *Rhodotorula mucilaginosa*, *Exophiala dermatitidis*, *Penicillium citrinum*, *Fomitopsis meliae*, and *Nectria pseudotrichia* identified at the collection site and *Candida spencermartinsiae* and *Penicillium chermesinum* at the city harbor. This study provides important insights into the fungal diversity associated with Amazon River dolphins and their environment, enhancing our understanding of the public health and ecological dynamics in the Anavilhanas National Park.

## 1. Introduction

Advancements in microbiology have improved the understanding of the microbiota of living animals. This enhanced understanding is important to study the complex relation animal-biota and for identifying potential zoonoses with potential risk to society [1].

Recent works have been describing the biota associated to animals, including cetaceans [1]. However, research specifically focusing on the Amazon River dolphins (*Inia geoffrensis*) is scarce. Among these limited studies, some have explored diseases affecting these dolphins. For example, cases of *Streptococcus iniae infection*, typically seen in captive dolphins, have been documented [2–4]. Furthermore, the occurrence of fungal pathogens, including those responsible for Jorge Lôbo's disease (JLD) or Lobomycosis, a chronic skin disease prevalent in humans in Central and South America, is of interest [5]. The causative agent of JLD is the fungus *Lacazia loboi* [6–9].

Conducting research on the skin microbiota and fungal pathogens of wild dolphins presents unique challenges. Gaining access to these animals and implementing suitable methodologies to describe the organisms present in their biota can be particularly daunting [10]. However, a unique opportunity for studying Amazon River dolphins has emerged at the Flutuante dos Botos, a private floating house situated in the Anavilhanas National Park in Novo Airão city, Amazonas state, Brazil [11, 12]. Since 1998, these dolphins have interacted with visitors and local residents at this location. This proximity offers a relatively easy access to conditioned Amazon River dolphin individuals and facilitates the collection of invaluable biological samples.

This study aims to fill the existing knowledge gaps concerning the fungal microbiota found in freshwater dolphins and leverage the potential for sample collection due to the distinctive interaction between these animals and humans in the city of Novo Airão-AM (Brazil). Specifically, our objectives are to collect skin and oral cavity samples from Amazon River dolphins, analyze the isolated fungi, and address the following research questions: (a) “what are the number of aerobic fungal colony-forming units (CFUs) present on the skin and in the oral cavity of the *Inia geoffrensis*?”; (b) “which fungal species are identified in the samples obtained from the skin and oral cavity?;” and (c) “are these fungal isolates previously documented in relation to infections in humans or these cetaceans?.” By providing answers to these questions, we aim to initiate a discussion about *Inia geoffrensis* microbiota and its potential implications.

## 2. Materials and Methods

### 2.1. Collection Site

**Figure 1:**
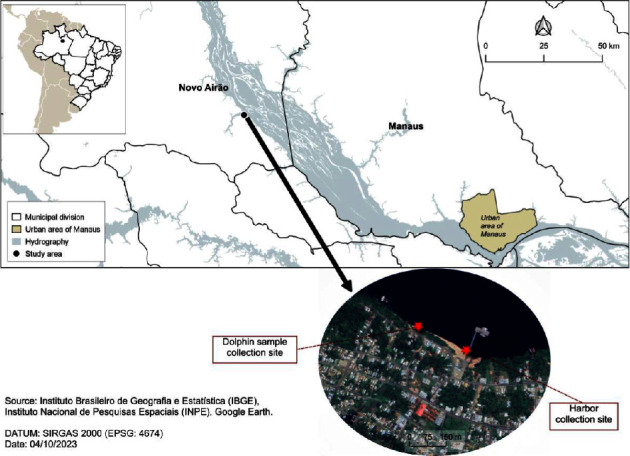
Sample collection locations for this work: City of Novo Airão. Dolphin sample (−2.60329, −60.945837), collection site (−2.59581, −60.94466), and harbor collection site (−2.61945, −60.94760). Software: QGIS, versão 3.18.10.

“Sample collections were carried out in the “Flutuante dos Botos” (a private floating house), where dolphins are provisioned on a daily basis and interact with visitors, and in the Novo Airão city harbor (Figure 1), both located in the south-central region of the Anavilhanas National Park, Amazonas state, Brazil. Novo Airão is a small town situated 183 km by land from Manaus and represents one of the main destinations for local inhabitants and tourists alike. The Anavilhanas National Park has approximately 350,000 hectares of area and about 400 islands, making it the second largest river archipelago in the world [10, 13, 14]. Its aquatic environment is formed by black waters of the Negro River, which are poor in nutrients and have high concentrations of organic compounds, resulting an acidic pH 4.6–4.8 while the dissolved oxygen level was 5.0–5.5 mg/L [15, 16]. It is important to note that compared to the Flutuante dos Botos, the city harbor of Novo Airão exhibited a higher degree and nature of pollution, influenced by boats pollutants influx and city sewage.”

### 2.2. Animals

The study involved the participation of three adult dolphins named Curumim, Chico, and Priscila. These dolphins (*Inia geoffrensis*) were visually identified with the assistance of the employees at Flutuante dos Botos, who possess expertise in recognizing individual dolphins based on their scars, pigmentation patterns, and behaviors [15, 16]. It should be noted that since these are free-ranging animals, estimating factors such as approximate age and sex becomes inherently challenging. The dolphins regularly partake in provisioning sessions at Flutuante dos Botos and willingly engaged in sample collection during the sessions conducted from June 6th to June 8th, 2022. During our visit to the facility, we observed that these animals did not exhibit any visible skin wounds; however, they did bear several scars, likely resulting from aggressive intraspecific interactions, previous trauma, and infections.

The research was carried out within the scope of the Chico Mendes Institute for Biodiversity Conservation (ICMBio), being authorized by the research committee of this institution and registered in the System of Authorization and Information on Biodiversity (SISBIO) under the numbers 37309-1 and 45110-1.

### 2.3. Sample Collection

**Figure 2:**
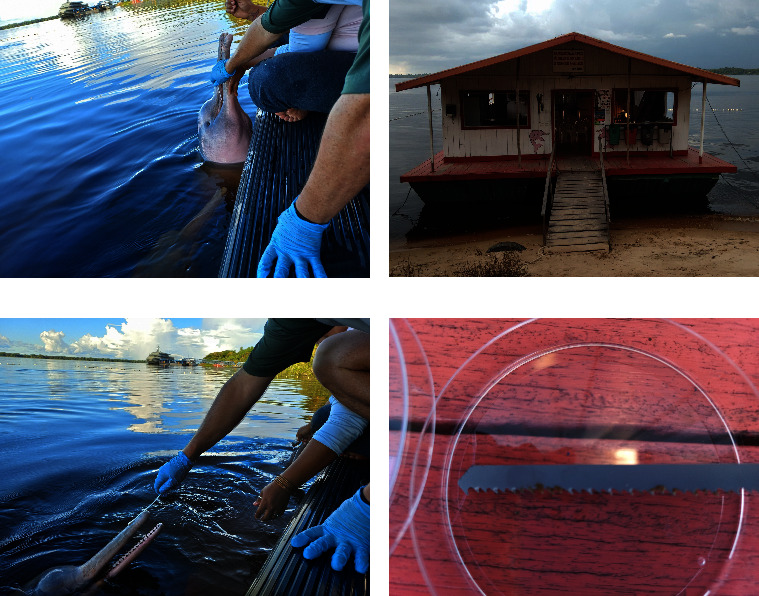
Images of sample collections (*Inia geoffrensis*). Collection of swabs from oral cavity from the boto “Curumim” (a); floating facility where the interaction with Amazon River dolphins occurred (b); collection of a skin sample from “Chico” (c); and small saw used to generate greater friction with the skin of dolphins (d).

To investigate the presence of fungi in both the skin and oral samples of dolphins (*I. geoffrensis*), we collected specimens and inoculated them immediately into culture media specifically designed for fungal isolation (Table 1). The dolphins voluntarily approached the floating deck surface, providing an opportunity for us to collect samples during their natural ascent.  Figure 2 displays images depicting the process of sample collection. For the collection of oral cavity and skin swabs, we utilized sterile disposable swabs PurFlock Ultra-6″ Sterile Standard Flock Swab w/Polystyrene Handle (Puritan Medical Products, Guilford, ME, USA). In our experimental conditions, these swabs keep about 100 ± 20 *μ*L of the biological specimens.

Swab from the oral cavity sample was collected from the interior region of the mouth, particularly from the space adjacent to and between the teeth, to accurately represent the oral microbiota. The skin samples were obtained from the dolphin's melon and rostrum.

In addition to the skin swab collection, we also collected a second sample from the dolphin's skin using a saw commonly employed in civil construction (Blade Single 75 × 3 mm, Bu38 Athol, Starrett Company, Massachusetts, United States) (Figure 1). During the dolphin's ascent, the saw was gently rubbed against the skin in the head region. The material obtained from the skin scraping was first transferred to the swab (PurFlock Ultra-6″ Sterile Standard Flock Swab w/Polystyrene Handle) and subsequently inoculated into the culture medium for further analysis.

With the purpose of investigating the fungi present in the water, we collected samples from the same collection site as the dolphins, approximately 30 cm deep, using sterile 25 mL conical tubes. These samples were transferred to the swab (PurFlock Ultra-6″ Sterile Standard Flock Swab w/Polystyrene Handle) and finally transferred to the culture media.

### 2.4. Fungi Isolation

In our experimental conditions, we assumed that each swab (PurFlock Ultra-6″ Sterile Standard Flock Swab w/Polystyrene Handle) transferred an approximate volume of 100 *μ*L or 100 mg of biological or environmental samples. These samples, one swab per sample and one swab for each culture media, were directly applied onto 9 cm plates containing the following five distinct culture media: Sabouraud agar, Mycosel, brain heart infusion, CHROMagar™ Candida (Becton, Dickinson and Company, USA), and niger seed agar.

The choice of these culture media was deliberate to isolate various fungi species, where Sabouraud agar supported medically relevant fungi, Brain heart infusion facilitated growth of dimorphic fungi, and CHROMagar™ Candida aided in the identification of *Candida* yeast species. In addition, the in-house prepared niger seed agar was specifically formulated for cultivating *Cryptococcus* genus agents. Following inoculation onto these media, plates were incubated at 25°C for 120 hours to optimize growth conditions. The composition of niger seed agar comprised *Guizotia abyssinica* (niger) seeds at 70 g/L, chloramphenicol at 0.05 g/L, agar at 20.0 g/L, and distilled water to achieve a final volume of 1000 mL, maintaining a pH of 6.7 ± 0.2 at 25°C [17, 18].

### 2.5. Fungi Identification

The fungi that developed were quantified and grouped into morphotypes and identified using conventional taxonomic keys [18, 19]. The morphological identification was associated with DNA sequencing analysis. Identification by DNA sequencing initiated with culture DNA was extracted with the kit QIAamp Tissue and Blood (Qiagen, Hilden, Germany) according to manufacturer. The PCR was performed in accordance with [20] from the amplification of the regions ITS (ITS1-5′ TCC GTA GGT GAA CCT GCGG 3′ and ITS4-5′ TCC TCC GCT TAT TGA TAT GC 3′). The following were used: 40 ng of DNA in a final volume of 30 *µ*L, with 1X reaction buffer (200 mM TrisHCl (pH 8.4), 500 mM KCl); 0.2 mM of each nucleotide; 2 mM of magnesium chloride (MgCl_2_); 50 ng of each primer; and 2.5U of Platinum® Taq polymerase enzyme (Invitrogen, Brazil). The amplicons were sequenced according to the instructions from the manufacturer of the kit “BigDye®Terminator v3.1 Cycle Sequencing Kit” (Applied Biosystems) and capillary electrophoresis was performed in an ABI 3130 Genetic Analyzer sequencer. The sequences were analyzed in the Sequencher 5.4.6 program and then compared with existing sequences in the NCBI database (https://blast.ncbi.nlm.nih.gov/Blast.cgi) and the Wester Dijk Fungal Biodiversity Institute (https://wi.knaw.nl/page/Pairwisealignment).

## 3. Results

The analysis, colony-forming units (CFUs), was conducted on three dolphins, named Curumim, Priscila, and Chico, from the Anavilhanas National Park, Novo Airão, Brazil. Various agar mediums, including Sabouraud, Chromagar Candida, agar niger, and brain heart infusion, were employed for fungal isolation (Table 1).

In Curumim, a single CFU of *Exophiala dermatitidis* was isolated from the oral swab using agar niger, with no fungal growth observed on skin swabs or scrapings. Priscila showed no fungal growth in any of the samples. However, in Chico, 3 CFUs of *Trichosporon montevi-deense* were identified in the oral swab, and 6 CFUs of *Toxicocladosporium irritans* along with an unidentified fungal colony were detected in skin scrapings.

In addition, water samples from the dolphins' habitat exhibited diverse fungal species, including *Rhodotorula mucilaginosa*, *Penicillium citrinum*, *Fomitopsis meliae*, *Exophiala dermatitidis*, and *Nectria pseudotrichia*. The water from the city harbor revealed over 100 CFUs of *Candida spencermartinsiae* and *Penicillium chermesinum*.

Brain heart infusion (BHI) agar was employed to detect dimorphic fungi. Although BHI agar is not selective for fungi, it revealed significant bacterial growth. In Curumim's samples, over 10 colony-forming units (CFUs) were recorded in the oral swab, skin swab, and skin scraping. A similar pattern was observed in Priscila's samples. Notably, Chico's samples and water samples exhibited an exceedingly high bacterial presence, with over 100 CFUs in all types of samples.

## 4. Discussion

In the Anavilhanas National Park, Brazil, we quantitatively and qualitatively assessed fungal species on Amazon River dolphins and in their aquatic environment. Through morphological analysis and sequencing of the ITS region, we identified diverse fungi, including *Trichosporon montevideense* and *Exophiala dermatitidis* from dolphin oral samples and *Toxicocladosporium irritans* from skin. Water samples revealed a higher fungal diversity, with significant findings such as *Rhodotorula mucilaginosa, Exophiala, Fomitopsis*, and *Penicillium citrinum*. This investigation enriches our understanding of the microbial landscape associated with these cetaceans and highlights potential implications for public health and ecosystem dynamics.

The lower fungal colony count in dolphins, as compared to their environment, points towards a robust immune system and a unique microbiota composition, potentially skewed towards bacterial dominance previous described [21, 22]. The fungi isolated from the water included *Penicillium*, *Rhodotorula*, and others belong to genera previously identified in water samples from the Rio Negro [23–25]. Predominantly from the phylum Ascomycota, these fungi are mostly transient in the environment, seeking suitable substrates for their growth and development [25].

The isolation of fungi from freshwater dolphins is scantily documented in literature [3, 22]. In our study, we detected *Trichosporon montevideense* and *Exophiala dermatitidis* in the oral cavity and *Toxicocladosporium irritans* from a skin sample. Though these genera are not known primary pathogens in dolphins, they hold significance in other contexts. *Trichosporon*, prevalent on mammalian skin, can cause skin infections such as onychomycosis and invasive infections in humans [18, 26]. *Exophiala dermatitidis*, an opportunistic fungus, can cause fatal brain infections and pheohyphomycosis in humans [27]. *Toxicocladosporium irritans*, isolated from dolphin “Chico,” have been found in patients with atopic dermatitis in Japan and in human blood and fingernails [28]. This investigation enriches our understanding of the skin microbiology of these cetaceans.

In the evaluation of zoonotic potential from cetacean oral microflora, it is crucial to consider the natural occurrence of these microorganisms within their habitat. The presence of diverse microbes in the oral cavities/skin of cetaceans, as observed through culture growth, does not inherently signify a pathogenic state. The low quantity of colony-forming units (CFUs) cultured suggests low potential pathological implications. Interestingly, according to our finds exposure to river water could pose a greater risk to fungal infection than the cetaceans themselves [18].

The present work has certain limitations that need to be acknowledged. These include the small number of animals and samples that were investigated. Due to these limitations, it was not possible to quantitatively evaluate the collection methods. To obtain a more comprehensive description of the microbiota, it is recommended that future experiments be conducted with a larger number of animals. However, it is important to consider the challenges involved in handling these animals. Moreover, the inability to collect samples beyond the designated ecotourism areas limited the comparative analysis of microbiota between dolphins engaged in ecotourism and those existing in non-tourism-associated habitats, signifying a potential constraint in drawing comprehensive conclusions regarding microbiota variations influenced by ecotourism activities. In addition, we believe that future work focusing on metagenomic studies will allow us to directly analyze the genetic material present in environmental samples. Unlike cultivable studies which rely on growing microorganisms in laboratory conditions, the metagenomic approach enables us to uncover the vast diversity of microorganisms, including non-cultivable species, and understand their functional potential and ecological roles. Nonetheless, this current work serves as a pioneering effort in describing the methodologies and presenting preliminary results. While providing foundational knowledge, it also opens avenues for further exploration into the complex interplay of environmental factors, microbial communities, and dolphin health.

## 5. Conclusions

In conclusion, our study has successfully addressed the revised research questions by providing a comprehensive analysis of the aerobic fungal colony-forming units (CFUs) present on the skin and in the oral cavity of Amazon River dolphins, identifying specific fungal species and assessing their potential implications for both dolphin health and human interaction. Our findings reveal the presence of *Trichosporon montevideense*, *Exophiala dermatitidis*, and *Toxicocladosporium irritans*, among others, highlighting a complex interplay between these dolphins and their microbial environment. Through these insights, we contribute to animal microbiology, emphasizing the need for integrated approaches that consider both animal and human health within ecologically sensitive habitats.

## Figures and Tables

**Table 1:** Analysis of aerobic fungal colony-forming units (CFUs) and species identification via PCR sequencing of ITS and 28S regions and micromorphology in the skin and oral cavity of Amazon river dolphins (*Inia geoffrensis*).

Sample		Colony forming unit/100 µL sample			
		Sabouraud	Chromagar candida	Niger seed	Brain heart infusion
					^*∗*^(Bacteria CFU)
Dolphin 1: “Curumim”	Oral swab	0	0	1 (*Exophiala dermatitidis*)	0 (>10)^*∗*^
	Skin swab	0	0	0	0 (>10)^*∗*^
	Skin scraping	0	0	0	0 (>10)^*∗*^

Dolphin 2:“Priscila”	Oral swab	0	0	0	0 (>10)^*∗*^
	Skin swab	0	0	0	0 (>10)^*∗*^
	Skin scraping	0	0	0	0 (>10)^*∗*^

Dolphin 3: “Chico”	Oral swab	0	3 (*Trichosporon montevideense*)	0	0 (>100)^*∗*^
	Skin swab	0	0	0	0 (>100)^*∗*^
	Skin scraping	6 (*Toxicocladosporium irritans*)	0	1 (*unidentified*)	0 (>100)^*∗*^

Water from the collection site		50 (*Rhodotorula mucilaginosa*)	2 (*Exophiala dermatitidis*)	1 (*Nectria pseudotrichia*)	0 (>100)^*∗*^
		1 (*Penicillium citrinum*)			
		1 (*Fomitopsis meliae*)			
		7 (*Exophiala dermatitidis*)			

Water from the city harbor		>100 (*Candida spencermartinsiae*)	1 (*Penicillium chermesinum*)	0	0 (>100)^*∗*^

*Note.* CFU (colony forming unit) denotes viable fungal colonies per 100 milliliter swab. Samples were collected from the oral cavities and skin of Amazon River dolphins identified by names given by the local community in the Anavilhanas National Park, Novo Airão–AM, Brazil. The agar types used were SAB (Sabouraud agar, Difco), CHRO (Chomagar Candida Bento Dickson), BHI (brain heart infusion agar, Difco), and NSA (niger agar). The samples are being presented with the “names given to the dolphins” by the community (Chico, Priscila, and Curumim).

## Data Availability

The data used to support the findings of this study are included within the article and supplementary materials (Table S1).

## Supplementary Materials

Supplementary Materials Table S1: “Fungal strains from Skin and Oral Cavity of Amazon River dolphins (*Inia geoffrensis*) and water samples in the Anavilhanas region, Amazonas: NCBI isolate accession numbers, % identity/similarity, and reference accession numbers.”
