# Supplementary material for: Cultivable Fungi from Amazon River Dolphins Engaged in Wildlife Ecotourism in the Anavilhanas National Park, Brazil
Source: Vet Med Int. 2024 Apr 23;2024:1267770. doi: 10.1155/2024/1267770 (PMC11074823; doi:10.1155/2024/1267770)
Supplement: Supplementary Materials — Table S1: “Fungal strains from Skin and Oral Cavity of Amazon River dolphins (Inia geoffrensis) and water samples in the Anavilhanas region, Amazonas: NCBI isolate accession numbers, % identity/similarity, and reference accession numbers.” [file 1267770.f1.docx]

Cultivable fungi from Amazon River dolphins engaged in wildlife ecotourism in the Anavilhanas National Park, Brazil

Marla J. Alves^1^, Fernanda Rodrigues Fonseca^2^, Layssa do Carmo Barroso^3^, Érica Simplício de Souza^4^, Marcelo Derzi Vidal^5^, Ani Beatriz Jackisch-Matsuura^1^, João Vicente Braga de Souza^6^*, Salvatore Siciliano^7^

Table S1 - "Isolated Fungal Strains from Bottlenose Dolphins and Water Samples in the Anavilhanas Region, Amazonas: NCBI Isolate Accession Numbers, % Identity/Similarity, and Reference Accession Numbers"

| Isolate | NCBI Isolate acession number | % Identity / similarity | References acession number |
| --- | --- | --- | --- |
| *Penicillium citrinum* A1-SAB_01 | [OQ918996](https://www.ncbi.nlm.nih.gov/nuccore/OQ918996) | NCBI (99,25%) / CBS-KNAW (99,32%) | KU897000.1 / CNRMA14.364 |
| *Fomitopsis meliae* A1-SAB_02 | [OQ919046](https://www.ncbi.nlm.nih.gov/nuccore/OQ919046) | NCBI (99,17%) / CBS-KNAW (98,78%) | KC585351.1 / SH1519482FU KR05775 |
| *Rhodotorula mucilaginosa* A1-SAB_03 | [OQ919155](https://www.ncbi.nlm.nih.gov/nuccore/OQ919155) | NCBI (99,63%) / CBS-KNAW (98,58%) | KP658860.1 / CBS 1382 |
| *Exophiala dermatitidis* A1-SAB_05 | [OQ919028](https://www.ncbi.nlm.nih.gov/nuccore/OQ919028) | NCBI (100%) / CBS-KNAW (99,21%) | MF320224.1 / SH1529581.08FU KX964623 |
| *Candida* *spencermartinsiae* A6-SAB_01 | [OQ919167](https://www.ncbi.nlm.nih.gov/nuccore/OQ919167) | NCBI (100%) / CBS-KNAW (99,14%) | OVV985326.1 / CBS10893 |
| *Penicillium* *chermesinum* A6-CHRO_01 | [OQ919247](https://www.ncbi.nlm.nih.gov/nuccore/OQ919247) | NCBI (100%) / CBS-KNAW (100%) | MN220649.1 / SH11605228FU KM278060 |
| *Trichosporon montevideense* A2-CHRO_01 | [OQ919245](https://www.ncbi.nlm.nih.gov/nuccore/OQ919245) | NCBI (95,12%) / CBS-KNAW (95,92%) | KP132872.2 / CNRMA8.1332 |
| *Diaporthe lithocarpus* A3-NSA_01 | [OQ923788](https://www.ncbi.nlm.nih.gov/nuccore/OQ923788) | NCBI (100%) / CBS-KNAW (96,02%) | KR703276.1 / SH1540604.08FU KU375690 |
| *Toxicocladosporium irritans* A7-SAB_01 | [OQ921702](https://www.ncbi.nlm.nih.gov/nuccore/OQ921702) | NCBI (99,26%) / CBS-KNAW (99,3%) | LN834443.1 / MH857249.1 / SH1572793.08FU JN974765 |
